# The Kinematics of Fixed-Seat Rowing: A Structured Synthesis

**DOI:** 10.3390/bioengineering10070774

**Published:** 2023-06-28

**Authors:** Tonio P. Agius, Dario Cerasola, Michael Gauci, Anabel Sciriha, Darren Sillato, Cynthia Formosa, Alfred Gatt, John Xerri de Caro, Robert Needham, Nachiappan Chockalingam, Joseph N. Grima

**Affiliations:** 1 Department of Physiotherapy, Faculty of Health Sciences, University of Malta, MSD 2080 Msida, Malta; tonio.p.agius@um.edu.mt (T.P.A.); anabel.sciriha@um.edu.mt (A.S.); john.xerri-decaro@um.edu.mt (J.X.d.C.); 2 Italian Rowing Federation, Viale Tiziano, 74, 00196 Rome, Italy; cerada@icloud.com; 3 Department of Psychology, Educational Science and Human Movement, University of Palermo, 90100 Palermo, Italy; 4 Metamaterials Unit, Faculty of Science, University of Malta, MSD 2080 Msida, Malta; michael.gauci.15@um.edu.mt; 5 Department of Podiatry, Faculty of Health Sciences, University of Malta, MSD 2080 Msida, Malta; darren.sillato.02@um.edu.mt (D.S.); cynthia.formosa@um.edu.mt (C.F.); alfred.gatt@um.edu.mt (A.G.); n.chockalingam@staffs.ac.uk (N.C.); 6 Centre for Biomechanics and Rehabilitation Technologies, School of Health, Science and Wellbeing, Staffordshire University, Stoke-on-Trent ST4 2DF, UK; r.needham@staffs.ac.uk; 7 Siġġiewi Rowing Club, 181, Melita Street, VLT 1129 Valletta, Malta

**Keywords:** biomechanics, kinematics, motion analysis, sport, rowing, fixed-seating rowing, coastal rowing

## Abstract

Olympic-style sliding-seat rowing is a sport that has been extensively researched, with studies investigating aspects related to the physiology, biomechanics, kinematics, and the performance of rowers. In contrast, studies on the more classic form of fixed-seat rowing are sparse. The aim of this study is to address this lacuna by analysing for the first time the specific kinematics of fixed-seat rowing as practised by able-bodied athletes, thus (i) documenting how this technique is performed in a manner that is replicable by others and (ii) showing how this technique compares and contrasts with the more standard sliding-seat technique. Fixed-seat rowing was replicated in a biomechanics laboratory where experienced fixed-seat rowers, marked with reflective markers following the modified Helen–Hayes model, were asked to row in a manner that mimics rowing on a fixed-seat boat. The findings from this study, complimented with data gathered through the observation of athletes rowing on water, were compared to sliding-seat ergometer rowing and other control experiments. The results show that, in fixed-seat rowing, there is more forward and backward thoracic movement than in sliding-seat rowing (75–77° vs. 44–52°, *p* < 0.0005). Tilting of the upper body stems was noted to result from rotations around the pelvis, as in sliding-seat rowing, rather than from spinal movements. The results also confirmed knee flexion in fixed-seat rowing with a range of motion of 30–35°. This is less pronounced than in standard-seat rowing, but not insignificant. These findings provide a biomechanical explanation as to why fixed-seat rowers do not have an increased risk of back injuries when compared with their sliding-seat counterparts. They also provide athletes, coaches, and related personnel with precise and detailed information of how fixed-seat rowing is performed so that they may formulate better and more specific evidence-based training programs to meliorate technique and performance.

## 1. Introduction

Olympic sliding-seat rowing is a sport that has been extensively researched and investigated from various aspects [1], including physiological parameters [2,3], psychological characteristics of the athletes [4,5], injuries [6,7,8], coordination [9,10], power output with an emphasis on the analysis of force/power curves [11], and athlete testing [12,13], as well as tactical strategies [14,15]. Other studies opted to focus on kinematics through measurements made [16,17,18] in the laboratory on ergometers and/or on water [19,20]. In contrast, however, the technique of fixed-seat rowing, a more classic form of the sport [21] that is still widely practised in various European countries, has been sparsely studied [22,23,24,25,26], and detailed knowledge about its kinematics acquired with modern instrumentation is limited to studies that focus on para-rowing [27].

**Figure 1:**
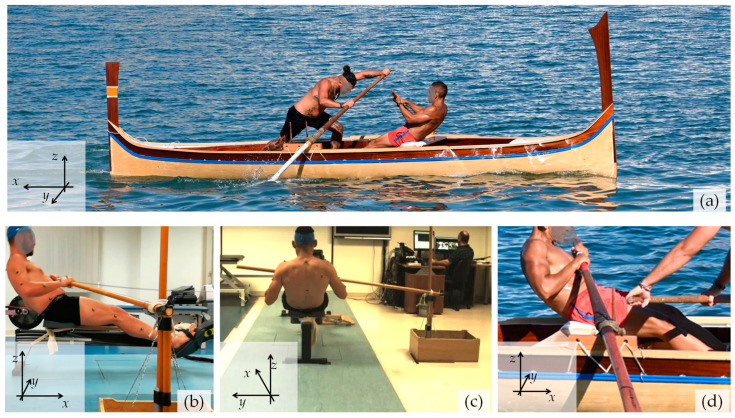
(**a**) The Maltese “Tal-Pass” boat, weighing 400 lbs, as rowed with two rowers, one sitting, locally referred to as *rmiġġ*, and one standing, locally referred to as *parasija*. (**b**,**c**) The setup used in the laboratory compared with (**d**), the actual on-water boat setup.

In fixed-seat rowing, athletes typically row the more traditional, wider and heavier, boats, generally suitable for coastal rowing, such as the Cornish Pilot Gig, Italian *galeoni*, or the Maltese *Dgħajsa tal-Pass*. The latter, a 400 lbs racing version of the traditional passenger boat, is the principal boat that has been used in the historical Maltese National Regatta for centuries and is rowed by a combination of seated and Venetian-style standing rowers [28] who row facing each other on the same boat, see Figure 1a, which can be rowed with two rowers as shown in Figure 1a, or four rowers; that is, two sitting, two standing. The technique of fixed seat-rowing adopted in competitions may vary slightly depending on the actual shape of the boat and the tradition followed. However, it is fairly standardised and generally practised with the rowers sitting on a static bench, fixed to the boat, with their legs pushing against static footrests.

The classic boat setups used in traditional rowing pre-date the introduction of the sliding-seat, which permits the rowers to efficiently generate power (known to be about 43–46% [10]) through the leg drive, in contrast with sliding-seat rowing, in which the contribution of the legs is considered to be less, as noted more than a century ago by Lehmann [21] and more recently by Izquierdo-Gabarren et al. [25]. In fact, recent work by Grima et al. [26] documented the prevalence of injuries in able-bodied fixed-seat rowers and reported that, compared with the standard sliding-seat rowers, fixed-seat rowers had a much lower incidence of knee injuries and a comparable incidence of back injuries.

As a result, the kinematics of fixed-seat rowing as practiced by able-bodied athletes, which *prima facie* is distinct to standard sliding-seat rowing, merits in depth analysis that extends beyond looking at it as the from used by para athletes, who row solely through a trunk and arm movement without using their legs (PR2 category, formerly referred to as TA or Trunk and Arms), or from the perspective of injuries [26] and performance indicators [22,23,24,25]. The purpose of this work is to address the important *lacuna* in the body of knowledge about fixed-seat rowing by analysing, using modern instrumentation in a controlled laboratory environment, the specific kinematics of fixed-seat rowing as practised by able-bodied athletes. More specifically, this work aims to (i) document this technique in detail in a manner that is replicable by others and (ii) assess how this technique compares and contrasts with the more standard sliding-seat technique.

## 2. Materials and Methods

Data collection was primarily carried out in a biomechanics laboratory setting, with data being recorded using calibrated motion-capture and analysis equipment driven via specialised software, which allowed for the information to be analysed qualitatively and quantitatively [28]. This was supplemented by on-water video analysis of pre-regatta rowing training, post-analysed manually primarily for qualitative or semi-quantitative purposes. Note that, throughout this manuscript, the standard rowing terms “drive”, “recovery”, “catch”, and “finish” shall be used [1]. When referring to on-water rowing, as graphically illustrated elsewhere [28], the following applies:

(a) The “drive” is the part of the rowing cycle with the oar in the water that propels the boat forward;
(b) The “catch” is the start of the drive and corresponds to when the oar catches the water and the pulling action of the oar in the water commences;
(c) The “finish” is when the rower removes the oar from the water at the end of the drive;
(d) The “recovery” is when the rower returns from the finish to the catch position with the oar outside the water.

### 2.1. Participants

Eight oarsmen, all male, age of 23.9 ± 2.6 years, height 177.6 ± 6.8 cm, and weight 89.1 ± 9.9 kg, who row in the Maltese National Regatta, volunteered to participate in this study. The inclusion criteria stated that rowers (i) had to have participated in at least two National Regattas; (ii) would have been rowing for at least three years in sitting positions with the oar on their right (known locally *rmiġġ*); (iii) had to be familiar with standard ergometer rowing; (iv) were above the age of 18; and (v) did not suffer from any acute musculoskeletal injuries or other morbidities that may preclude inclusion, e.g., cardiorespiratory problems. These rowers were informed about the procedure verbally and through a detailed information letter. All participants signed an informed consent form that was previously approved by the Faculty Research and Ethics Committee (approval number: FREC FHS_1718_017). The procedure was non-invasive and posed no different risk than those undertaken by the rowers during ergometer training. Rowers were free to choose which experiment they wished to perform and be analysed for.

### 2.2. Laboratory Setup and Equipment Used

The laboratory-based study was carried out in the Biomechanics Research Laboratory at the Faculty of Health Sciences, University of Malta. This windowless laboratory is permanently setup with a Vicon motion-capture system (Oxford metrics, Oxford, UK), which can be used to perform motion analysis at a 100 Hz sample rate, with ambient conditions kept constant in a controlled manner, as demanded by scientific rigour.

The experiments carried out were designed to replicate fixed-seat rowing in sweep form, as practised in Malta, with the oar always being on the right-hand side of the rower (*rmiġġ*). Malta was chosen as the location for this investigation because it offered the researchers a practically unique test population, that is, subjects who only practised and competed on fixed-seat boats, using the fixed-seat rowing technique, which has remained unchanged for many generations.

Data collection was carried out using a standard Concept2 Model D rowing ergometer, which was modified so that the seat would remain fixed (i.e., not permitted to slide), and the ergometer plastic handle was replaced with a wooden rod-like handle, meant to replicate an oar handle as used in Malta. As shown in Figure 1, this wooden handle was held by the rower at one of its ends, as if it was a real oar, and affixed near its other end using a rope to a fixed vertical pin, in a manner that replicates the normal oarlock used on the Maltese boats. Additional sets of control experiments were also carried out using the aforementioned handle, with the seat allowed to slide (experiment CE1); using the standard plastic handle supplied with the Concept2 rowing ergometer with the seat not permitted to slide (experiment CE2); as well as with the Concept2 rowing ergometer as supplied by the manufacturer, i.e., with no modifications in the handle and with the seat allowed to slide (experiment CE3). For ease of comparison and reference, these four modalities shall be referred as Modality I, II, III, and IV, respectively, as in Table 1. For the purpose of this work, the rowing ergometer was arbitrarily assigned an orientation in space such that the floor corresponds to the *xy*-plane, as shown in Figure 1.

### 2.3. Protocol

The rowers were marked with reflective markers following the modified Helen–Hayes model (Plug-in-Gait, Vicon^®^, Oxford, UK [29,30]) and were asked to warm up in their preferred manner. Each participant was asked to row on the ergometer (Concept 2) or modifications of it, as described in Table 1, at a self-selected pace, in terms of both stroke rate and power, and their movements were recorded over four 10 s captures. The captures were performed at randomly selected periods during the trial. Rest periods were not allowed in between captures to ensure a steady rowing rate. However, when the same rower participated in more than one experiment, adequate rest time was given, also permitting for change of setup.

The position of the markers was detected using the 16-camera setup, the capture envelope was defined, and the cameras were calibrated using a standard calibration protocol that ensured the best accuracy possible. During the data-capture process, the Vicon Nexus^®^ software version 2.8.1 (Oxford metrics, Oxford, UK) recognised the coordinates for each of the 39 spherical reflective markers for every camera in the setup, which were used to generate a set of angular measurements related to the joints performing the movements. These angles define movements of the thorax, pelvis, spine, hip, knee, angle, shoulders, and elbows in the sagittal, coronal, and transverse planes (henceforth referred to as planes 1, 2, and 3, respectively) and were measured with the individual sitting on the rowing ergometer as in Figure 1.

Note that, for the purpose of this work, the following important angular measurements *θ*, which define the kinematics for this type of exercise, were measured and analysed (see Supplementary Information for further details):

- Thorax1 and Pelvis1, which measure the anterior/forward (+ve) or posterior/backward (−ve) tilt of the thorax and pelvis, respectively;
- Spine1, which relates the measured forward (+ve) or backward (−ve) thorax and pelvis tilts, relative to each other;
- Hip1, which measures the flexion or extension of the lower limb forward or backwards;
- Knee1, which measures the flexion or extension around the knee joint axis, measured relative to the hip, between the thigh and the shank;
- Ankle1, which measures the dorsiflexion around the tibia *y*-axis, the angles between the shank and the foot;
- The Shoulder1, Shoulder2, and Shoulder3 angles, measured in three planes relative to the thorax, where
  (i) Shoulder1 measures flexion or extension where, for a person standing straight, the arm would be moving forward and backwards;
  (ii) Shoulder2 measures abduction or adduction where, for a person standing straight, the arm would be moving up or down in a pure sideways direction;
  (iii) Shoulder3 measures rotation around the axis of the humerus;
- Elbow1, which measures the flexion or extension of the arm around the elbow joint axis.

### 2.4. Data Analysis

Three full cycles (strokes), from finish to finish, were selected and, from each, the range of motion *θ*_ROM_ associated with the angular measurement *θ* was recorded as the difference between *θ*_max_, the maximum value of the angle *θ* attained within the cycle, and *θ*_min_, the minimum value of the angle *θ* attained within the cycle.

All angular measurements obtained *θ* were plotted as a percentage of the cycle, where the data were modulated in such a manner that the ‘catch’ and ‘finish’ events were synchronised for all of the recorded cycles for each experiment, in order to obtain an appropriately averaged cycle of measurements for each of the four experiments (Table 1).

Inferential statistics [31] were computed using the Statistical Package for Social Science (SPSS^®^) software, version 25, using the Mann–Whitney test to identify statistical differences when comparing any two different modalities through a comparison of *θ*_ROM_, *θ*_min_, and *θ*_max_. A *p*-value less than or equal to 0.05 (*p* ≤ 0.05) indicates that, at a 95% confidence level, the differences are probably not due to sampling error, i.e., that the angular measurements are probably different.

### 2.5. Additional On-Water Data Capture

In addition to the laboratory captures, a video analysis of on-water kinematics was also conducted during training periods in the three weeks preceding a National Regatta. For the purpose of this work, only rowers who row with their oar on the right-hand side were studied (*rmiġġ*), and they were assigned to the modality M-V, as defined in Table 1. These data were recorded and post-analysed as described in the Supplementary Information.

## 3. Results

**Figure 2:**
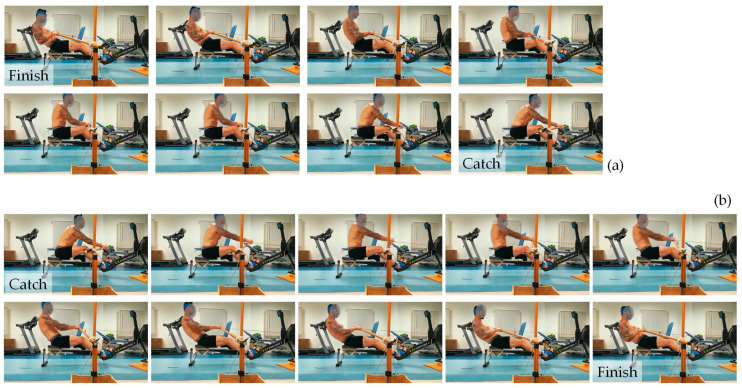
A detailed visual representation of (**a**) the recovery phase, from finish to catch, and (**b**) the drive phase, from catch to finish, in fixed seat rowing as emulated by the experiment in the lab (M-I). The images from the slower recovery phase are taken at half the frame rate of the drive phase.

**Figure 3:**
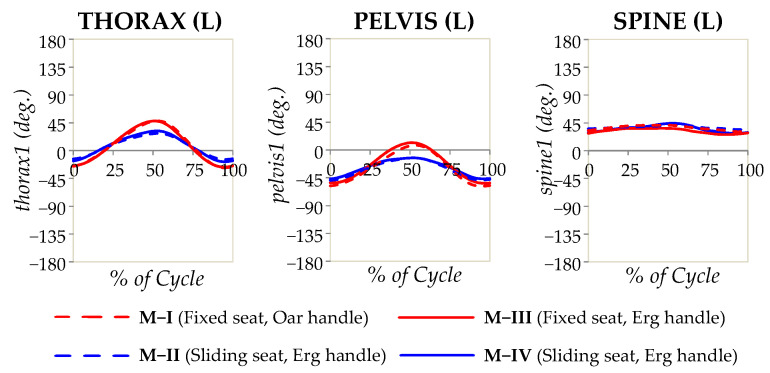
The angular measurements for the thorax, pelvis, and spine in the sagittal plane (Thorax1, Pelvis1, and Spine1) plotted as a percentage of the rowing cycle, as recorded from the laboratory study. The solid lines refer to experiments where the standard Concept2 ergometer handle was used, while the broken lines refer to experiments where the oar replica was used, with red corresponding to fixed-seat and blue corresponding to sliding-seat.

**Figure 4:**
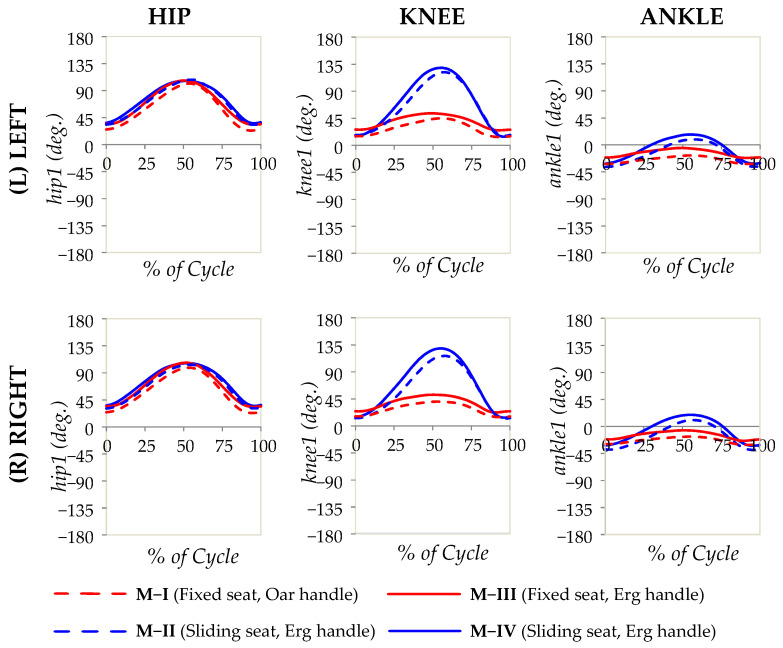
The angular measurements for the hips, knees, and ankles in the sagittal plane (hip1, knee1, and ankle1) plotted as a percentage of the rowing cycle, as recorded from the laboratory study. The solid lines refer to experiments where the standard Concept2 ergometer handle was used, while the broken lines refer to experiments where the oar replica was used, with red corresponding to fixed-seat and blue corresponding to sliding-seat.

**Figure 5:**
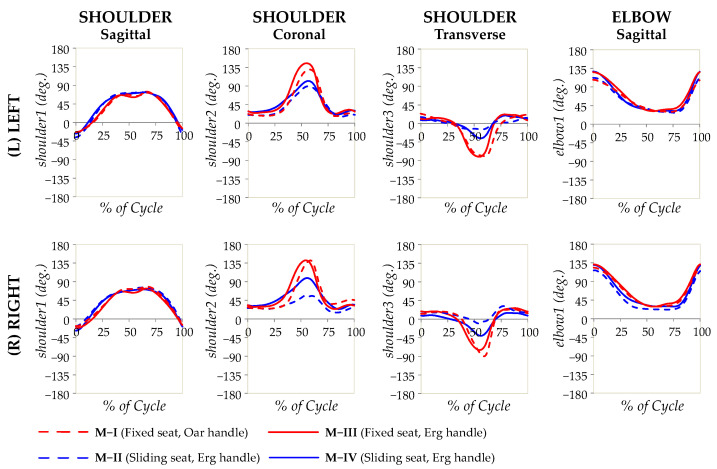
The angular measurements for the shoulders and elbow plotted as a percentage of the rowing cycle, as recorded from the laboratory study. The solid lines refer to experiments where the standard Concept2 ergometer handle was used, while the broken lines refer to experiments where the oar replica was used, with red corresponding to fixed-seat and blue corresponding to sliding-seat.

**Figure 6:**
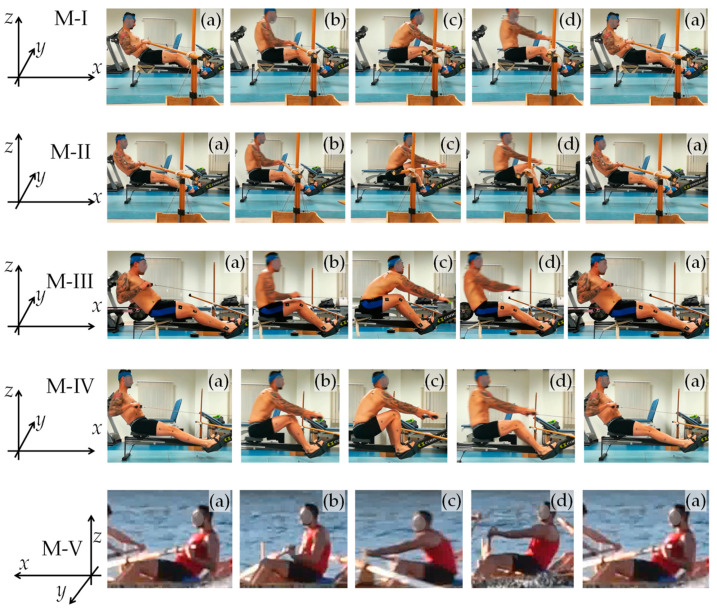
Images from the different modalities studied in the lab (M-I, M-II, M-III, and M-IV), showing one full cycle, from finish to finish, compared to each other and to on-water (M-V). In these images, a cycle is represented by five distinct images, labelled (**a**–**d**), where (**a**) represents the finish, (**b**) represents approximately mid-recovery, (**c**) represents the catch, and (**d**) represents approximately mid-drive.

A set of consecutive photographic images that show a typical subject undergoing the main experiment (M-I) at various stages of the rowing cycle is shown in Figure 2. Note that the “recovery” phase corresponds to Figure 2a and the “drive” phase corresponds to Figure 2b. Moreover, the angular measurements taken in the laboratory are reported as graphs showing a variation in the angular measurements with a percentage of the cycle, finish to finish, in Figure 3 (thorax, pelvis, and spine), Figure 4 (hips, knees, and ankles), and Figure 5 (shoulders and elbows). The mean and standard deviations of *θ*_ROM_ are reported in Table 2 together with the computed *p*-values when the ranges of motion for M-I are compared to those of M-II, M-III, and M-IV. The values of *θ*_min_ and *θ*_max_ and the *p*-values that correspond to *θ*_min_ and *θ*_max_ are reported in the Supplementary Information. To help with the interpretation of these measurements, one may refer to Figure 6, which shows a pictorial representation of all modalities, where the finish (0% and 100% on the graphs) corresponds to images marked as ‘a’, while the catch corresponds to images marked as ‘c’. Note that M-I and M-V correspond to the main technique being studied (fixed-seat sweep rowing), with M-I being performed in the lab while M-V is performed on water.

The Supplementary Information also includes the following:

(1) Re-plots of Figure 3, Figure 4 and Figure 5 to include information of the spread of measurements (average ± 1.96 standard deviations), see Figures S1–S3;
(2) Traced shapes of the back profiles of various rowers while rowing fixed-seat on water on Maltese traditional fixed-seat racing boats (M-V), shown in Figure S4, represented alongside of the equivalent profiles captured during standard sliding-seat rowing on the ergometer (M-IV);
(3) Estimates of the extent of knee flexion made from video analysis of on-water rowing (Figure S5).

Note that, owing to the inevitable limitations associated with on-water measurements, these on-water measurements should only be treated as semi-quantitative estimates that are meant to supplement the laboratory measurements.

These results suggest, in a visual and quantitative manner, that the kinematics of fixed-seat rowing is distinct from that of sliding-seat rowing even if there are various features that are common to both. The key findings include (i) an appreciably greater upward and downward tilt of the upper body compared with when the sliding-seat is used, which seems to stem from rotations around the pelvis; (ii) an observable knee flexion in fixed-seat rowing, albeit this is less pronounced than in sliding-seat rowing; and (iii) a more complex pattern of shoulder movements. Note also that, in the form of traditional rowing studied here, as evident in Figure 1, Figure 2 and Figure 6, the sitting rower is holding the oar with a “reverse grip” [32], rather than the “normal grip” used in standard sweep rowing, where the palm of the outer hand faces up and the palm of the inner hand faces down.

## 4. Discussion

### 4.1. Biomechanics and Kinematics of the Thorax, Pelvis, and Spine

As evident from the results, thoracic motion mainly happens in the sagittal plane, a movement that was found to be dependent on whether or not a sliding-seat is used. This finding is driven by the necessity to elongate the drive length and may be explained though the following observations:

i. Rowers lean more posteriorly at the finish when using fixed-seat as opposed to sliding-seat: the minimum angles (which occur when ‘finishing’) with fixed-seat (M-I and M-III) are around −28°, with these being lower when using sliding-seat, at −15° to −20° (M-II and M-IV);
ii. The rowers are inclined further forward at the catch when rowing fixed-seat as opposed to sliding-seat: the maximum angles (which occur around the ‘catch’) with fixed-seat (M-I and M-III) are *c.* +49°, with these being considerably greater than when using sliding-seat, at *c.* +30° (M-II and M-IV).

The net result is a more pronounced forward and backward motion of the thoracic region when rowing fixed-seat (75–77°) than sliding-seat (44–52°). This difference was supported by the analysis, which indicates no difference between M-I and M-III (both fixed seat), but when comparing fixed-seat vs. sliding-seat (i.e., M-I vs. M-II, M-IV), there was a measurable and significantly different thoracic profile of movement (see plots), with *p* for $\theta_{X}\in \left\{\theta_{max},\theta_{min,\;}\;\theta_{ROM}\right\}$ for M-I vs. M-II and M-I vs. M-IV always being less than 0.0005. This finding is congruent with the findings of Cutler et al. [27], who studied simulated PR2 (TA) para-rowing, which is effectively fixed-seat, with the authors reporting ‘a significantly greater range of lumbar motion than that reported in the able-bodied literature by increasing the joint angle not only at the catch position preferentially but also at the finish position’. This greater range of movement, when put in context of the fact that it is not atypical for fixed-seat rowers to row at mildly higher stroke rates compared with their sliding-seat counterparts to make up for the shorter drive length, would be reflected in higher peaks in the angular velocity for fixed-seat vs. sliding-seats, very steep slopes in the velocity curves at the catch and finish, and higher acceleration and deceleration movements by the rowers to increase the range of movement of their upper body compared with sliding-seat in the shorter time period of one stroke.

Focusing on the manner in which the thoracic plane behaves in relation to the pelvic plane, it is important to note that a comparison of the thorax and pelvic plots (Figure 3) suggests that these two segments, both measured relative to the ground, follow each other, suggesting that the thorax angle is dictated by the orientation of the pelvic plane. In fact, for all seated modalities, thoracic movement is always a slight augmentation of that happening in the pelvis, with this minor augmentation being provided through the slight movement of the lumbar spine, measured in this case through the ‘Spine’ measurements. An interesting finding is that the present work suggests that the spine movements in the sagittal plane in M-I, fixed-seat rowing with oar, is actually less than that for M-IV, standard sliding seat ergometer rowing (14.6° for M-I vs. 17.3°), albeit this difference is not statistically significant (*p* = 0.254). However, there was a difference between M-I and M-II, where the range of values was 9.3° (*p* = 0.024), but these values are not as dissimilar between modalities as were those of the thorax or pelvis angles.

These findings effectively dispel the common idea that, in fixed-seat rowing, there is much greater reliance on the spine to achieve movement when compared with sliding-seat rowing, as the present work clearly indicates that the highly noticeable apparent flexion of the upper body in fixed-seat rowing, measurable through the thorax angles relative to the ground, is shown to be primarily the result of pelvic movements. This very important finding, deduced from the laboratory setting, is confirmed through the observation of back profiles for on-water rowers (see Supplementary Information), which clearly indicates that, despite the much wider range of lumbar motion associated with fixed-seat rowing as opposed to standard Concept2 sliding-seat rowing (M-IV), there is very little visible change in the curvature of the back in the lumbar region. More importantly, this finding provides a biomechanical explanation to our recent finding that fixed-seat and sliding-seat rowers experience the same incidence rate of back injuries [26]. It must be emphasised that the pelvic movement described here is the result of movement of the hip going into flexion through the action of muscles such as iliopsoas and rectus femoris as prime movers of the hip joint. Thus, even when the pelvis is stabilised, as in PR2 (TA) para-rowing, there would still be a degree of movement into hip flexion. Such a finding is also evident in Cutler’s work [27], which looked at fixed-seat rowing performed by able-bodied rowers, where the athletes are seated on a fixed-seat with the feet held by straps, effectively being in a closed kinetic chain.

An observation must be made of pelvic movements in modalities M-I and M-III (fixed seat), which, as a result of the manner in which the human body moves, feature an inevitable degree of anteversion and retroversion owing to the seat being fixed. The pelvis must, by necessity, perform these movements to allow for hip and spinal mobility. Were this not the case, the tendency for increased stresses on the lower lumbar spine would be inevitable [27]. Thus, one may hypothesise that fixed-seat rowing may be particularly beneficial from a therapeutic perspective for athletes who need to stabilise their pelvic muscles and lower back. This observation may also carry some implications with reference to para-rowing, which should be further investigated by researchers in this field, to ensure that any strapping applied to PR2 rowers (who are only obliged to strap their knees) is not applied in a manner that may possibly limit pelvic mobility unnecessarily, thus leading to increased stresses on the lower back during the rowing cycle.

### 4.2. Biomechanics and Kinematics of the Knee, Ankle, and Hip of Sitting Rowers

Through this study, other important findings related to knee movements in the sagittal plane can be deduced. The first finding is that, in contrast to common perception, and the historical manner in which fixed-seat rowing used to be instructed [21], there is a non-insignificant degree of knee flexion. This is clear from the graphs, which suggest that there is a range of motion of *c.* 30°, a movement that, for all seated modalities, at the finish sees the knee in the position of terminal extension at knee angles of *c.* 15°. Here, it must be noted that this range of motion for the knee from 15° at the finish to *c.* 45°–50° at the catch has been observed in the lab for M-I and M-III, the two fixed-seat modalities, and observed in on-water analysis, as clearly evident from the plots in Figure 6, which confirms that, for the various forms of fixed-seat Maltese boats, there is always an appreciable and visible movement at the knee joint for seated rowers in the fixed-seat boats studied.

Obviously, although the degree of knee flexion is not negligible, this is not as much as one observes in sliding-seat rowing, as evident from the plots and the *p*-values < 0.0005 when comparing M-I to M-II/M-IV. Nevertheless, it still follows the same pattern in the sense that it flexes at the catch and extends at the finish, indicating that the rowers in fixed seat boats are still using, to some extent, the powerful leg muscles. However, given the negligible incidence of knee injuries caused by fixed rowing [26], compared with standard sliding-seat rowing [6,7,8,33,34,35], it is evident that such leg usage is much less taxing on the athlete. It is also encouraging that the work reported here for M-IV is also in agreement with Sforza’s findings [32].

The knee movements are also reflected in the ankle measurements, which follow a similar profile as the feet remain in contact with the footplate (or its equivalent on the boat). This implies that, in both fixed- and sliding-seat seated rowing, an element of dorsiflexion and plantar flexion at the ankle occurs, a movement that is greater for modalities M-II and M-IV compared with M-I (and M-III). Flexion is greatest at the catch, i.e., when the knee is flexed.

Moving up to the hip, the most remarkable aspect associated with this movement is the observation that rowers in the fixed-seat modality using an oar replica exhibited the widest, but generally not too dissimilar, range of motion in the hip in the sagittal plane. Most importantly, there is no discernible difference in the sagittal movements comparing M-I vs. M-II, left and right. In the case of M-I vs. M-IV, left and right, it is interesting to note that the value of *p* < 0.05 corresponds to the range of motion for M-I being even higher than that for M-IV. All of this clearly suggests that fixed-seat rowing still utilises the full range of hip movement as used by their sliding-seat counterparts, even if the knee and pelvic movements are not equivalent.

### 4.3. Biomechanics and Kinematics of the Shoulder and Elbow of Sitting Rowers

A comparison of shoulder profiles between modalities reveals some very interesting characteristics, rendering the shoulder kinematics in seated fixed-seat rowing very distinct to that of sliding-seat rowing. Here, one should make reference to a statement made by Nolte [1], that the body of the rower should be considered as the link in the chain between the blade and the foot stretcher, i.e., all of the power and movement generated through the knee and swinging at the pelvis needs to be transmitted to the blade via the shoulders and upper limbs in the drive phase. Moreover, as is common knowledge in the field of rowing (advocated more than a century ago by Lehmann [21]), in the recovery phase in sliding-seat rowing, shoulders are meant be kept in a relaxed state and not ‘unnaturally stiffened’ and there must only be a passive ‘unconscious movement’ in the forward direction (shoulder flexion). At the same time, it must also be recognised that, irrespective of how the rower is moving, the aim of the rowing action is to pull the oar with as much power as possible during the drive phase. This is considerably facilitated by using strong lower limbs in sliding-seat rowing. In the case of fixed-seat rowing, the rower needs to rely more on the upper body to generate the required power to compensate for the reduced movements at the knee joint. These factors are probably the origin of some important differences between shoulder movements in fixed-seat and sliding-seat rowing. In fact, when one compares the averaged range of motion in M-II and even more in M-IV (sliding-seat ergometer rowing), one can observe a single peak within the cycle, just after the catch, whereas in fixed-seat rowing, there are two very distinct peaks, with one occurring just before the catch and another in preparation for it. This first ‘pre-catch’ peak can be compared to a deadlift weightlifting exercise, where the shoulder muscles are ‘cocked’ just before to initiation to take up the slack of the bar and weight when lifting. This, in essence, is an activation of the rotator cuff and the latissimus dorsi muscles to enable an efficient catch. What is being reported here is consistent with observations by Smoljanović et al. [11]. These authors noted a tendency to maximise stroke lengths in order to optimise performance in PR1 (AS) class rowers, para-athletes who are *de facto* a special distinct category of fixed-seat rowers. These rowers predominantly row with their shoulders and arms, while being strapped around the mid-thorax to provide stability and back support, and effectively also eliminating any pelvis movements, by ‘reaching over with their shoulders and upper back’.

A distinction also arises in the higher shoulder range of motion observed in the coronal plane, meaning that, in fixed-seat rowing, there is much more pronounced shoulder abduction. This is primarily the result of a much wider abduction at the finish (the maximum abduction angle), where the present study observes maximum abduction angles of around 135° at the catch for the fixed-seat modalities, as opposed to only around 90° for the sliding-seat modalities. Here, it should be noted that Cutler et al. [27] also noted a significant increase in shoulder abduction angles when comparing PR2 (TA) to PR3 (formerly referred to as LTA or Legs, Trunk, and Arms para athletes, who can make use of their legs to move a sliding-seat) and PR1 (AS) to PR2 (TA), a feature that the authors state to be present at both the catch and finish, with the PR2 (TA) setup being the closest to what is studied here. However, in contrast to Cutler et al. [27], the present work (1) did not identify a statistically significant difference in minimum abduction angles associated with the finish and (2) observed a much wider range of motion than that observed by Cutler et al. [27] in their PR2 (TA) setup. These differences between the present work and that of Cutler et al. [27] are possibly because the present cohort was more accustomed to fixed-seat rowing when compared with sliding-seat rowing, as opposed to participants recruited by Cutler et al. [27]. Thus, it is to be expected that the present cohort would have learnt to maximise the efficiency of the fixed-seat rowing action via shoulder abduction. The present work highlights this aspect of shoulder abduction even more than what Cutler et al. [27] report, and thus one can now undeniably confirm the claim that shoulder abduction is a unique characteristic of fixed-seat rowers, who tend to maximise the efficiency of the rowing cycle in this manner. In other words, it should not be merely considered as “a unique feature to para-rowing setups, and should be investigated in more depth as it relates to the design of the fixed-seat and strapping used in TA and AS rowing” [27], but a common feature in fixed-seat rowing.

In the case of the elbow, the present work also identifies an increased range of motion in the elbow, if one had to compare M-I to M-IV (*p* = 0.004 for the left elbow and *p* < 0.0005 for the right elbow). However, this difference is mostly related to the use of a sweep oar rather than a standard ergometer handle, whether rowing in a fixed- or sliding-seat manner.

### 4.4. Strengths and Limitations of This Work

This work looked into the biomechanics of traditional fixed-seat rowing as practised by able-bodied athletes from a kinematics perspective and managed to identify a number of aspects that so far had never been addressed. The main strengths of this work are as follows:

i. What is being studied here represents ‘virgin territory’ from a research perspective because the cohort of athletes that were studied were all athletes who had never rowed on, or experienced, sliding-seat boats prior to data collection;
ii. Several aspects related to the technique of fixed-seat rowing that were either previously ignored or never formally recorded or studied have now been identified;
iii. The methodology used was based on experiments performed in a state-of-the-art calibrated laboratory setting that replicated traditional fixed-seat rowing rather than data collected on site with the associated limitations in taking on-water measurements;
iv. What was studied here, with the help of participants who had always rowed fixed-seat, could shed light on what PR1 and PR2 para-rowers, who, like the cohort of athletes studied here, have always rowed fixed-seat, actually experience. It also suggests that certain aspects of how para-rowing is conducted may need to be further examined. These relate to the manner in which athletes are strapped to the boat, which has the benefit of providing stability, but could be causing unintentional discomfort by physically prohibiting all forms of movements of the legs.

Unfortunately, the study also had a few limitations. In particular:

i. This work only looked at kinematic aspects, with no measurements being made either of muscle activity (which could have been done through standard methods such as EMG) or by looking at forces, which could have been measured using additional equipment such as load-cells to measure the load through the chain and other equipment that could have analysed other forces such as bending of the oars;
ii. The rowers were permitted to row at their own pace, which resulted in data generation that needed to be processed quite extensively in order to make the catch and finish points coincide;
iii. No attempt was made to standardise the data apart from aligning the ‘catch’ and ‘finish’. It is well known that gait analysis, height, and age are determinants of stride length and cadence. Therefore, researchers such as Scrutton [36], Kirtley, Whittle et al. [37], and O’Malley [38] suggested and implemented normalisation using mathematical formulae to exclude height from being a prime determinant of gait analysis, especially in the paediatric field, where height variation is very broad according to age (it is worth noting that one of the studies addressed children between 2 and 14 years). In the present case, such a normalisation was not possible because of the fact that, at this very preliminary stage of work, it is not yet clear which are the most important parameters to standardise against and the present sample size is too small. In view of this limitation, the original data are being reported in full in the relevant appendices in the hope that, in the future, if more data are collected, the present data can be re-analysed and adequately normalised.
iv. The cohort of athletes studied could have been larger to improve the statistical significance, and it would have been ideal to link the technique to the results obtained. Unfortunately, both of these limitations were not easy to avoid. The latter aspect of linking the technique to the results obtained, while sounding easy, may have its own ethical issues given that the Maltese cohort of athletes is so small.

## 5. Conclusions

This work has studied, for the first time, the kinematics of seated fixed-seat rowing, as performed by able-bodied athletes on traditional boats, in a scientific approach through combined laboratory-based and on-site work. This has led to a number of important observations related to rowing movements associated with this classic form of the sport. In particular, the following was shown:

1. There is highly noticeable apparent flexion of the upper body in fixed-seat seated rowing, confirmed through the thorax angle measurements relative to the ground, which were found to be primarily the result of pelvic/hip movements and not flexion of the lower back, as previously assumed by many through casual observation;
2. In fixed-seat rowing, there is an appreciable movement at the knee joint for seated rowers, a finding confirmed quantitatively through the laboratory measurements, as well as through the post-hoc analysis of rowing movements on various fixed-seat boats. These knee movements were not as pronounced as in sliding-seat rowing, but still present.
3. While movement of the shoulder angles in sliding-seat rowing, when averaged, is observed, to a first approximation, to only peak once within the cycle, just after the catch, in fixed-seat, two very distinct peaks appear, with an additional peak occurring just before the catch and in preparation for it.

These findings have helped provide the needed biomechanical explanation for the observed injuries experienced by fixed-seat rowers, who seem to be at no increased risk of lower back injuries compared with their sliding-seat counterparts. Moreover, this work, for the first time, provides athletes, coaches, and specialists with the toolkit that is necessary to properly understand how competitive fixed-seat rowing is performed, and will hopefully provide an impetus to further studies on this classic form of rowing, which is increasing in popularity.

## Figures and Tables

**Table 1:** A brief summary of the characteristics of the four modalities studied in the laboratory and the one studied on water.

Key	Location	Experiment	Handle/Oar	Seat Type
M-I	Lab	Main experiment (EXP)	Wooden oar replica	Fixed seat
M-II	Lab	Control (CE1)	Wooden oar replica	Sliding seat
M-III	Lab	Control (CE2)	Standard erg plastic handle	Fixed seat
M-IV	Lab	Control (CE1)	Standard erg plastic handle	Sliding seat
M-V	On water	Real scenario	Actual wooden oar	Fixed seat

**Table 2:** A comparison of the range of motion in degrees for the angular measurements made.

		Range of Motion *θ*_ROM_ (deg.) Mean ± Standard Deviation				*p*-Values		
		M-I	M-II	M-III	M-IV	M-I vs. II	M-I vs. III	M-I vs. IV
Seat Type: Handle/Oar		Fixed Seat Oar	Sliding Seat Oar	Fixed Seat C2 Handle	Sliding Seat C2 Handle			
Thorax1	L	77.5 ± 12.3	44.4 ± 7.2	75.8 ± 8.2	52 ± 11.3	<0.0005	0.722	<0.0005
Pelvis1	L	67.9 ± 7.7	37.3 ± 8.1	67.2 ± 8	36.3 ± 9.3	<0.0005	0.866	<0.0005
Spine1	L	14.6 ± 6.1	9.3 ± 2.6	13.8 ± 2.8	17.3 ± 6.6	0.024	0.707	0.254
Hip1	L	80.2 ± 11.8	75.9 ± 12.1	75.3 ± 10.9	72.5 ± 9.9	0.113	0.115	0.016
	R	77.5 ± 11.2	74.1 ± 11.4	75.5 ± 9.8	72.4 ± 10.2	0.441	0.389	0.064
Knee1	L	32.9 ± 11.3	107.7 ± 14.5	29.7 ± 14.3	116.6 ± 13.1	<0.0005	0.321	<0.0005
	R	27.7 ± 8	107 ± 17	30.8 ± 9.7	116.8 ± 14.6	<0.0005	0.379	<0.0005
Ankle1	L	15.9 ± 4.8	45.9 ± 3.1	17.5 ± 7.4	49.4 ± 5.8	<0.0005	0.822	<0.0005
	R	15 ± 3.7	51.2 ± 4	18.2 ± 8.4	52.7 ± 6.3	<0.0005	0.252	<0.0005
Shoulder1	L	101.1 ± 11.9	109.3 ± 6.4	99.3 ± 10.6	101.5 ± 7.9	0.098	0.694	0.982
	R	99.5 ± 11	95.3 ± 8.2	97.6 ± 11.2	100.6 ± 9.7	0.153	0.349	0.793
Shoulder2	L	120.2 ± 19.4	80.2 ± 22.5	127.9 ± 9.8	86 ± 27.7	<0.0005	0.144	0.001
	R	143.5 ± 56.5	57.2 ± 16.3	121.3 ± 17.4	80.2 ± 21.8	<0.0005	0.224	<0.0005
Shoulder3	L	112.2 ± 36.1	48.8 ± 15.7	109.1 ± 22	62.4 ± 28.9	<0.0005	0.694	<0.0005
	R	123.5 ± 34.1	50.4 ± 13.3	107.8 ± 34.6	63.6 ± 29.7	<0.0005	0.134	<0.0005
Elbow1	L	95.7 ± 7.7	95.4 ± 6.8	103.7 ± 10	102.4 ± 6.3	0.892	0.030	0.004
	R	79 ± 17	85.1 ± 7.8	96.1 ± 9.1	98 ± 6.7	0.634	0.004	<0.0005

## Data Availability

Data are contained within the article and Supplementary Information.

## Supplementary Materials

The following supporting information can be downloaded at: https://www.mdpi.com/article/10.3390/bioengineering10070774/s1, S1: Protocol for On-Water Data Collection and Analysis; S2: Results related to Laboratory Measurements and definition of Angles Measured; S3: Results related to On-Water Measurements.
