# Supplementary material for: The Kinematics of Fixed-Seat Rowing: A Structured Synthesis
Source: Bioengineering (Basel). 2023 Jun 28;10(7):774. doi: 10.3390/bioengineering10070774 (PMC10376464; doi:10.3390/bioengineering10070774)
Supplement: Supplementary file 1 [file bioengineering-10-00774-s001.zip › bioengineering-2420454-supplementary.pdf]

SUPPLIMENTARY INFORMATION

THE KINEMATICS OF FIXED-SEAT ROWING:

A STRUCTURED SYNTHESIS

*Tonio P. Agius[1], Dario Cerasola[2,3], Michael Gauci[4], Anabel Sciriha[1], Darren Sillato[5], Cynthia Formosa[5,6], Alfred Gatt[5,6], John Xerri de Caro[1], Robert Needham[6], Nachiappan Chockalingam[5,6], Joseph N. Grima [4,7] **

(1) Department of Physiotherapy, Faculty of Health Sciences, University of Malta, Msida, MSD 2080, Malta.

(2) Italian Rowing Federation, Viale Tiziano, 74, 00196, Rome, Italy.

(3) Department of Psychology, Educational Science and Human Movement, University of Palermo, 90100, Palermo, Italy.

(4) Metamaterials Unit, Faculty of Science, University of Malta, Msida, MSD 2080, Malta.

(5) Department of Podiatry, Faculty of Health Sciences, University of Malta, Msida, MSD 2080, Malta.

(6) Centre for Biomechanics and Rehabilitation Technologies, School of Health, Science and Wellbeing, Staffordshire University, Stoke-on-Trent, ST4 2DF, United Kingdom.

(7) Siġġiewi Rowing Club, 181, Melita Street, Valletta VLT 1129, Malta.

** Corresponding Author. e-mail: joseph.grima@um.edu.mt*

S1: Protocol for On-Water Data Collection and Analysis

S1.1 Data Capture

The recording was carried out from the vantage point which is approximately around ¾ along the way of the normal training and racing route, with cameras mounted on tripods, located around 2-3 metres above sea-level. A point perpendicular to the motion being carried out was chosen in order to eliminate any perspective or parallax errors and only that part of the recording in which the boat was at this specific point was utilised for analysis purposes. The recording of rowers who had their oar on the right was done with the camera facing their left side. Recording was carried out in a manner which did not interfere whatsoever with the rowers' training to the extent that they were not even aware that they were being filmed to negate the well-known Hawthorne and Rosenthal effects.

Video recording was performed using a Nikon D3200 DSLR camera with a Sigma lens (150-600mm) and a Nikon DSLR Camera D750, with a Sigma 70-200 f/2.8 in the three weeks preceding a National Regatta. Recording was performed at a high enough zoom-level where the individuals were clearly and sharply identifiable, ensuring that around 2-4 complete cycles were available to have at least one cycle for analysis.

S1.2 Data Extraction

All recordings were transferred to a computer in their original format and manually filtered in order to choose the captures which were suitable for analysis based on the quality of the video and the time of capture. Quality and usability of a video were considered if complete, non-blurred cycles could be identified, with the boat being as perpendicular to the camera-visualisation-line as possible. The selected cycles were 'catch-to-catch', for identification purposes, as judged from the position of the seated rower in the M-V position, as this was found to be the optimal, identifiable position. The videos chosen were edited, splitting the video using the 'Movavi' video-editing software (www.movavi.com). Images were extracted from the respective clip at a frame rate of 18 frames per cycle. Key positions which defined the posture and position of the rowers were identified manually and highlighted by placing individually labelled small circular markers for each of these key points. The coordinates of these key points were then identified through an in-house written script which outputs the pixel numbers of selected positions. This procedure was preferred over an automated method as there was not enough contrast between the location and the rest of the image, to enable automatic detection of these key locations via the software used. This was due to a combination of factors including:

i. the fact that the subjects were not marked in any manner for ease of identification;
ii. the presence of inappropriate direct lighting conditions, since training was carried out outdoors with the availability of only natural light and the time of day leading to the formation of shadows thus rendering visualisation of pre-attached markers practically impossible since the time of practice was usually late afternoon to early evening;
iii. the athletes rowed with different amounts of clothing that might have precluded the visualisation of joint location;
iv. although the cameras and lenses were of relatively high quality, resolution might have been altered due to the ambient light conditions and the zoom levels utilised;
v. the boats are inherently unstable in character, due to ambient conditions, such that the relative position of the rower relative to the camera capturing the movement on a fixed location, was subject to random deviations in yaw, roll and pitch due to the ambient weather/water conditions;
vi. as a result, the zoom level affected the resolution of the captures together with the fact that the cameras were possibly subject to a level of judder due to the windy conditions in the area of the harbour where the filming was taking place.

The coordinates, in terms of pixel numbers of the extracted key points were used to obtain a measure of the back curvature and to compute the various angular measurements required, the most important of which was the sagittal knee angle (i.e. knee1 as defined above).

S2: Results related to Laboratory Measurements and definition of Angles Measured

(a) Definition of Angles

The protocol used from the data-capture process in the laboratory is based on the Plug-in-Gait model as implemented within the Vicon Nexus® software version 2.8.1 (Oxford metrics, Oxford, United Kingdom) which generates a set of angular measurements related to the joints performing the movements. These angles are normally measured with the individual standing parallel to the vertical z-direction and facing the *x*-laboratory direction (the direction of normal walking), or in this specific case, *mutatis mutanda*, sitting on the rowing ergometer as in Figure 1. The manner how these angles are defined and calculated are explained in detail in the software reference guide which details the "Plug-in Gait output angles" (https://docs.vicon.com/display/Nexus215/Plug-in+Gait+output+angles).

More specifically, the following angles are being reported in this work:

(i) Angles measuring the orientation of the thorax and pelvis, reported as absolute angles relative to the laboratory axis, where:

- *Thorax1 measured the backwards or forward tilt about the lab y-axis;*
- *Pelvis1 measured anterior (+ve) or posterior (-ve) tilts around the lab y-axis;*

(ii) The spine angles which relate with the measurements of the aforementioned thorax and pelvis angles, relative to each other, where:

- *Spine1 measured forward (+ve) or backward (-ve) thorax tilt around the pelvic y-axis;*

(iii) The shoulder angles, which were measured relative to the thorax, where:

- *Shoulder1 measured flexion around the thoracic y-axis;*
- *Shoulder2 measured abduction around the (shifted) thoracic x-axis*
- *Shoulder3 measured internal rotation around the (doubly shifted) thoracic z-axis*

(iv) The elbow angles, where:

- *Elbow1 measured flexion around the humeral axis*

(v) The hip angles, which are measured relative to the pelvis, where:

- *Hip1 measured flexion around the pelvic y-axis;*

(vi) The knee angles were measured relative to the hip, measuring the angles between the thigh and the shank and included:

- *Knee1 measured flexion around the thigh y-axis;*

(vii) The ankle angles, i.e. the angles between the shank and the foot, were:

- *Ankle1 measured dorsiflexion around the tibia y-axis.*

(b) Results

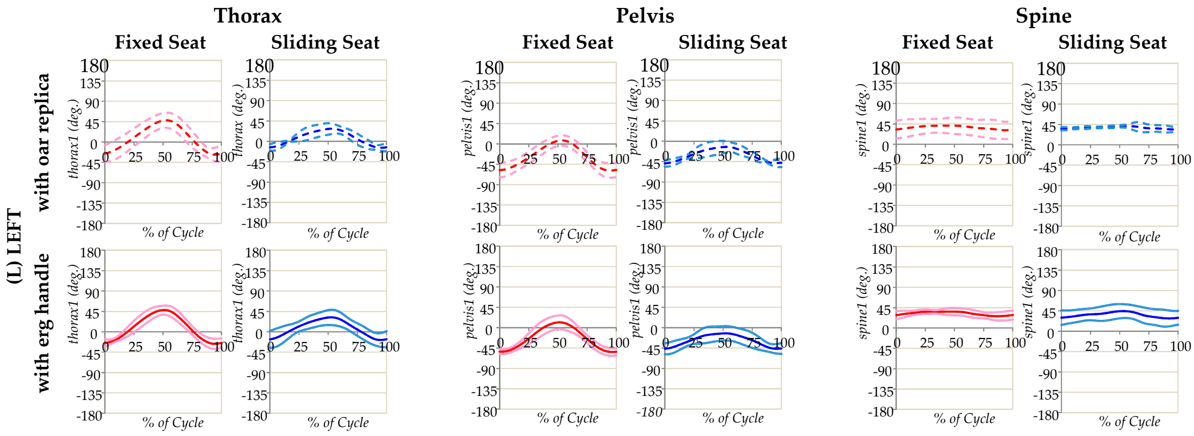

Figure S1: The angular measurements for the thorax, pelvis and spine in the sagittal plane (Thorax1, Pelvis1 and Spine1) plotted as a percentage of the rowing cycle as recorded from the laboratory study. The darker lines represent the average whilst the lighter ones represent the region of ±1.96 standard deviations. The solid lines refer to experiments where the standard Concept2 ergometer handle was used whilst the broken lines refer to experiments where the oar replica was used with red corresponding to fixed-seat whilst blue corresponds to sliding-seat.

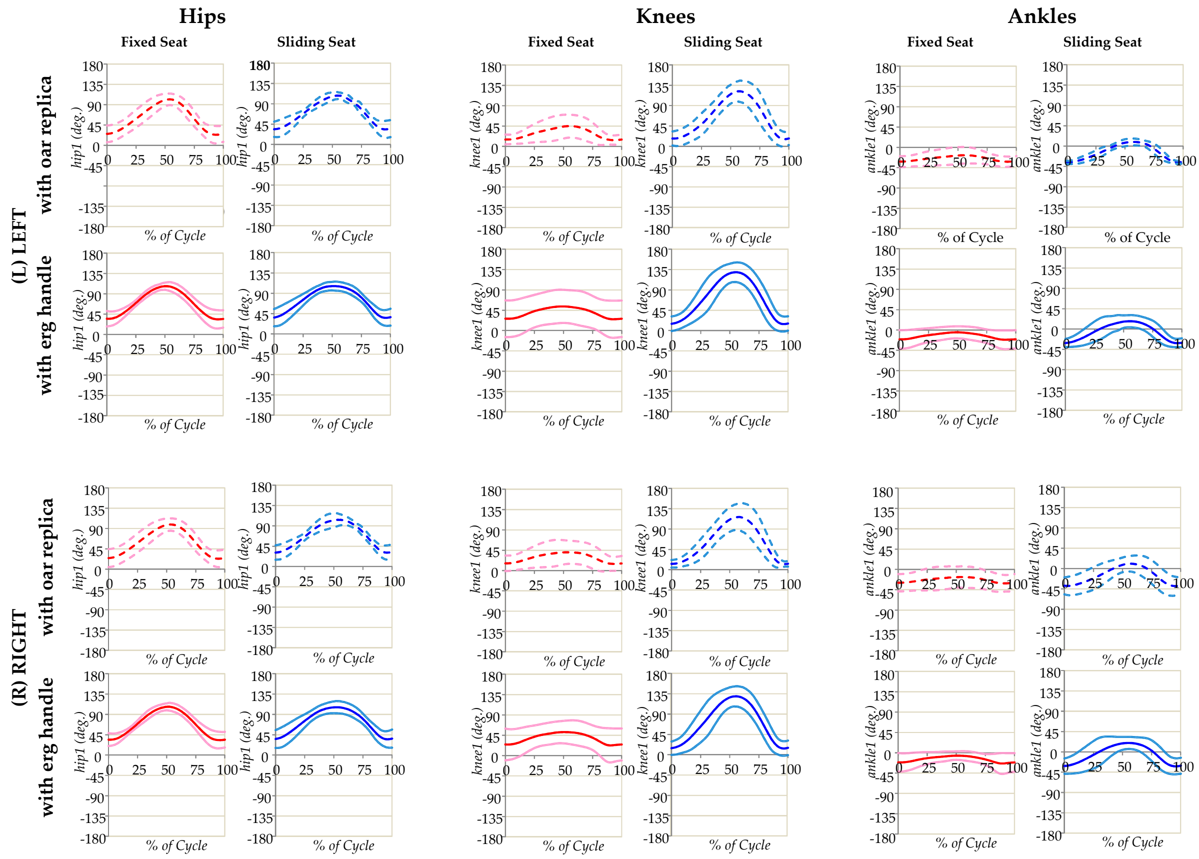

Figure S2: The angular measurements for the hips, knees and ankles in the sagittal plane (hip1, knee1 and ankle1) plotted as a percentage of the rowing cycle as recorded from the laboratory study. The darker lines represent the average whilst the lighter ones represent the region of ±1.96 standard deviations. The solid lines refer to experiments where the standard Concept2 ergometer handle was used whilst the broken lines refer to experiments where the oar replica was used with red corresponding to fixed-seat whilst blue corresponds to sliding-seat.

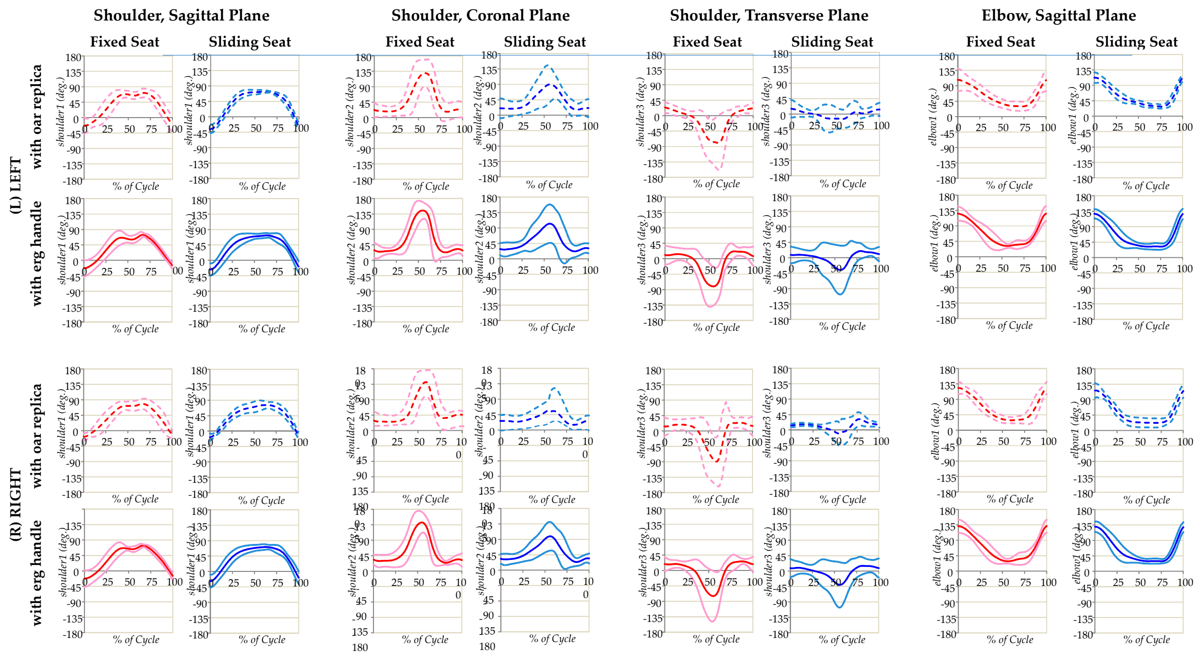

Figure S3: The angular measurements for the shoulders and elbow plotted as a percentage of the rowing cycle as recorded from the laboratory study. The darker lines represent the average whilst the lighter ones represent the region of ±1.96 standard deviations. The solid lines refer to experiments where the standard Concept2 ergometer handle was used whilst the broken lines refer to experiments where the oar replica was used with red corresponding to fixed-seat whilst blue corresponds to sliding-seat.

Table S1

		Minimum Angular Value θ_{min} (deg.)				p-Values		
		**M-I**	**M-II**	**M-III**	**M-IV**	**M-I *vs.* II**	**M-I *vs.* III**	**M-I *vs.* IV**
Seat Type: Handle/Oar		*Fixed seat Oar*	*Sliding Seat Oar*	*Fixed seat C2 Handle*	*Sliding seat C2 Handle*			
Thorax1	L	-28.8 ± 7.6	-15.6 ± 2.5	-27.3 ± 5	-20 ± 7.6	0.000	0.851	0.000
Pelvis1	L	-59.8 ± 7.4	-49.4 ± 3.1	-55.1 ± 3.9	-48.3 ± 4.7	0.000	0.081	0.000
Spine1	L	29.1 ± 8.7	33.4 ± 3.1	24.9 ± 4.3	27.4 ± 8.6	0.838	0.048	0.624
Hip1	L	22.1 ± 8.8	32.9 ± 9.2	32 ± 9.8	34.5 ± 8.7	0.004	0.035	0.041
	R	21.8 ± 8.7	30.6 ± 8.3	31.9 ± 8.7	33.9 ± 9.2	0.057	0.002	0.005
Knee1	L	12.4 ± 5.4	15.3 ± 7.3	24.1 ± 20.8	13.1 ± 8.4	0.000	0.171	0.000
	R	13.3 ± 8.2	11.4 ± 2.7	22.1 ± 18.2	12.9 ± 7.8	0.000	0.051	0.000
Ankle1	L	-33.1 ± 6.1	-36.8 ± 1.7	-22.5 ± 10.8	-32 ± 4.4	0.000	0.004	0.000
	R	-31.5 ± 10.2	-39.8 ± 10.6	-24 ± 11.7	-33 ± 9.5	0.000	0.023	0.000
Shoulder1	L	-25.8 ± 8.7	-34.8 ± 6	-23.3 ± 11.9	-26.7 ± 9	0.539	0.679	0.758
	R	-17.9 ± 5.1	-17.1 ± 4.4	-23.1 ± 11.5	-27.9 ± 9.3	0.036	0.001	0.000
Shoulder2	L	12 ± 8.3	14.3 ± 9.9	18.2 ± 6.1	18.8 ± 7.7	0.001	0.025	0.002
	R	13.4 ± 10.7	11.1 ± 12.3	21.4 ± 3.6	21 ± 5.9	0.000	0.421	0.000
Shoulder3	L	-88 ± 34.5	-25.8 ± 15.5	-82.5 ± 29.2	-38.6 ± 34.6	0.683	0.368	0.846
	R	-94.9 ± 37.7	-16.6 ± 16.8	-76.5 ± 37.7	-42.7 ± 32.6	0.204	0.476	0.049
Elbow1	L	28.9 ± 4.2	21.9 ± 7	28.8 ± 3.2	28 ± 3.6	0.039	0.051	0.014
	R	29 ± 6.4	27.4 ± 2.8	30.9 ± 3.1	30.5 ± 4.3	0.401	0.000	0.000

Table S2

		Maximum Angular Value θ_{max} (deg.)				p-Values		
		**M-I**	**M-II**	**M-III**	**M-IV**	**M-I *vs.* II**	**M-I *vs.* III**	**M-I *vs.* IV**
Seat Type: Handle/Oar		*Fixed seat Oar*	*Sliding Seat Oar*	*Fixed seat C2 Handle*	*Sliding seat C2 Handle*			
Thorax1	L	77.5 ± 12.3	44.4 ± 7.2	75.8 ± 8.2	52 ± 11.3	0.000	0.599	0.001
Pelvis1	L	67.9 ± 7.7	37.3 ± 8.1	67.2 ± 8	36.3 ± 9.3	0.001	0.045	0.000
Spine1	L	14.6 ± 6.1	9.3 ± 2.6	13.8 ± 2.8	17.3 ± 6.6	0.221	0.149	0.524
Hip1	L	102.3 ± 6.4	108.7 ± 3.6	107.2 ± 3.7	106.8 ± 4.8	0.008	0.009	0.000
	R	99.4 ± 6.8	104.4 ± 6.6	107.3 ± 3.9	106.3 ± 6.9	0.014	0.016	0.000
Knee1	L	20.9 ± 15.8	11.8 ± 13.9	31.8 ± 7.6	25.4 ± 15.9	0.467	0.229	0.891
	R	45.2 ± 12.8	122.7 ± 12	53.8 ± 18.8	129.7 ± 11	0.307	0.421	0.546
Ankle1	L	41 ± 13.2	118.4 ± 16.3	52.7 ± 12.8	129.7 ± 11.4	0.093	0.005	0.758
	R	-17.3 ± 9.2	9.3 ± 3.8	-5 ± 6.8	17.5 ± 6.8	0.031	0.252	0.327
Shoulder1	L	75.3 ± 5.2	74.3 ± 2	76.1 ± 2.8	75 ± 3.4	0.017	0.216	0.546
	R	81.7 ± 7.8	78.3 ± 5.6	74.5 ± 2.4	72.7 ± 3.8	0.909	0.301	0.000
Shoulder2	L	132.3 ± 19.5	94.6 ± 25.7	146 ± 12.7	104.6 ± 28.1	0.352	0.045	0.035
	R	156.9 ± 55.7	68.3 ± 18.7	142.8 ± 15.8	101.2 ± 20.5	0.734	0.014	0.008
Shoulder3	L	24.2 ± 7.2	23.2 ± 6.9	26.6 ± 9.5	24 ± 9.3	0.000	0.574	0.000
	R	28.7 ± 9.6	33.8 ± 9.9	31.3 ± 6.3	21 ± 11.2	0.000	0.184	0.000
Elbow1	L	124.5 ± 8.3	117.2 ± 9.6	132.2 ± 10.2	130.3 ± 7.2	0.018	0.327	0.216
	R	107.9 ± 16	112.4 ± 7.5	126.8 ± 10.1	128.5 ± 6.7	0.400	0.175	0.278

S3: Results related to On-Water Measurements

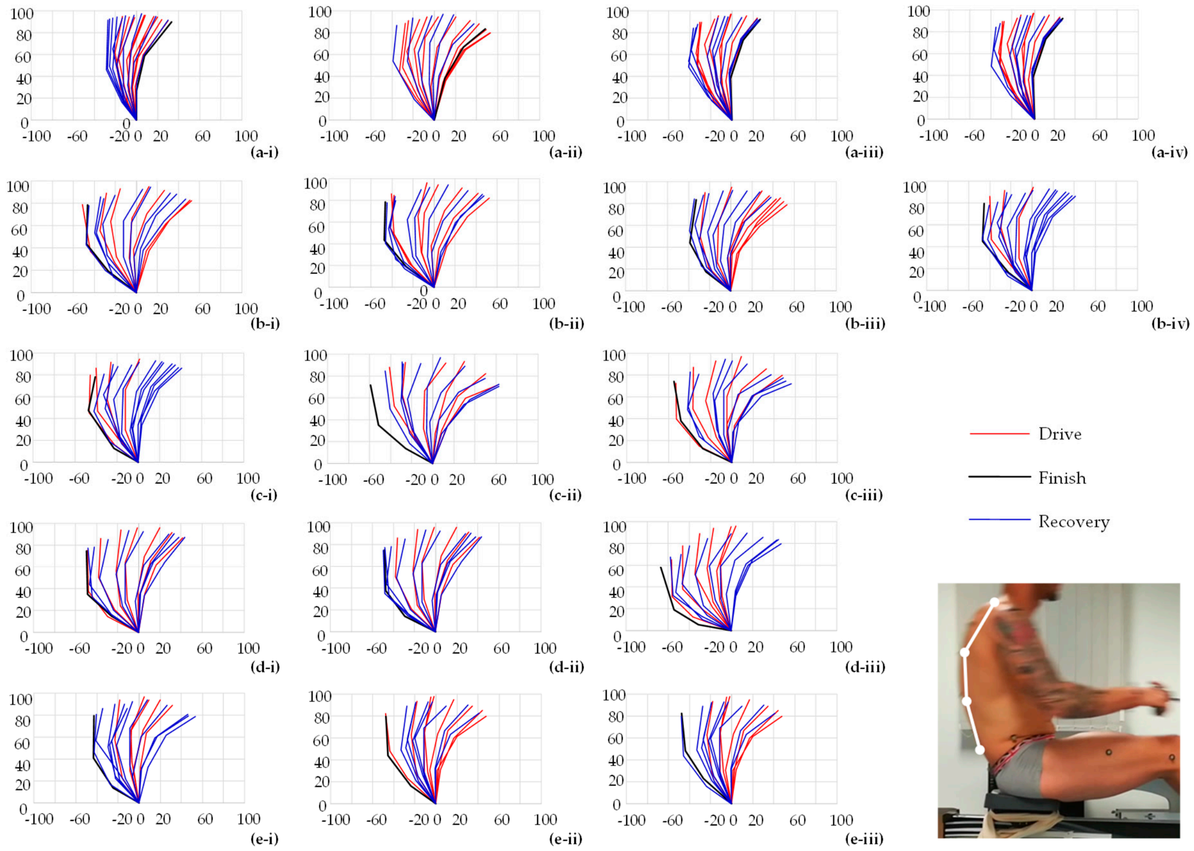

Figure S4. Images showing the typical outline of the back profiles traced from images extracted from video, where (a) shows four cycles of rowers using a standard sliding-seat rowing ergometer, whilst (b), (c), (d) and (e) refer to the equivalent profiles from rowers rowing on different types of boats. The boats studied are all traditional Maltese rowing racing boats where (b) is the widest and heaviest *Tal-Pass* boat which can be rowed by two rowers, one standing one sitting (b-i and b-ii), or four rowers, two standing, two sitting (b-iii and b-iv); (c) refers to a slightly longer, more slender and lighter *Tal-Medalji* version, also rowed by four rowers, two standing, two sitting; (d) refers to the much shorter *Kajjik*, rowed by rowers, one standing, one sitting; and (e) refers to the slowest and shortest boat, the *Frejgatina* rowed by two seated rower with a coxswain. All these boats are rowed in the traditional Maltese National Regattas. The rower analysed is always the one seated with the oar on his right-hand side (*rmiġġ*). The manner how these profiles were drawn is illustrated in the insert.

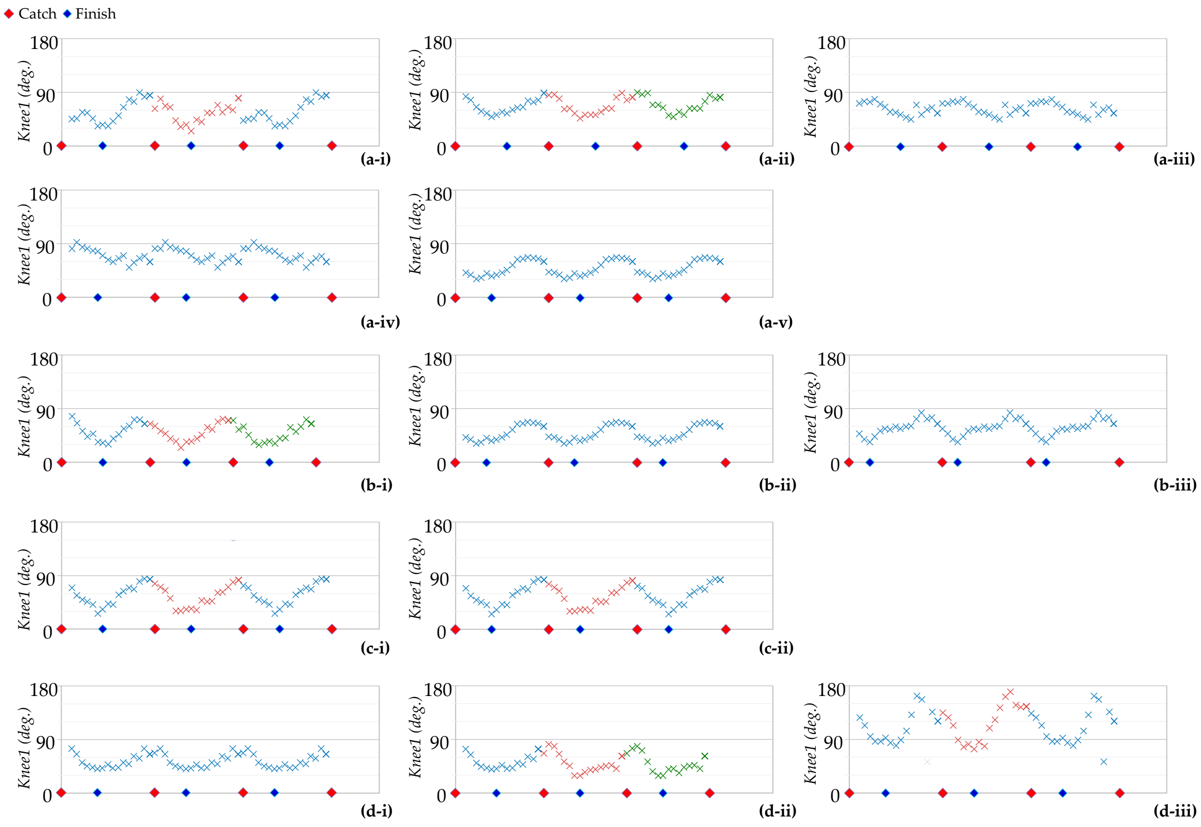

Figure S5. Estimates of knee flexion, sagittal movements, shown as three cycles, as observed in the actual on-water competitive seated fixed-seat rowing for the different boats studied where (a) refers to the widest and heaviest *Tal-Pass* boat rowed by two rowers (i-iii) or four rowers (iv,v); (b) refers to the slightly longer, more slender and lighter *Tal-Medalji* version; (c) refers to the much shorter *Kajjik* and (d) refers to the slowest and shortest boat. The rower analysed is always the one seated with the oar on his right-hand side (*irmiġġ*). The "catch" and "finish" are indicated on the *x*-axis, where different colours of data-points correspond to different cycles by the same rower.
